# Immune Evasion by Neurotropic Viruses: Molecular Strategies, Cellular Targets, and Consequences for CNS Infection

**DOI:** 10.3390/ijms27177962

**Published:** 2026-09-07

**Authors:** Antonios Mouzakis, Vasileios Petrakis, Katerina Chlichlia

**Affiliations:** 1 Laboratory of Molecular Immunology, Department of Molecular Biology and Genetics, Democritus University of Thrace, 68100 Alexandroupolis, Greece; antonios.n.mouzakis@gmail.com; 2 Department of Infectious Diseases, Second Department of Internal Medicine, University General Hospital Alexandroupolis, Democritus University of Thrace, 68100 Alexandroupolis, Greece; vpetraki@med.duth.gr

**Keywords:** neurotropic viruses, central nervous system (CNS), immune evasion, viral persistence, neuron-glia interactions

## Abstract

Neurotropic viruses have evolved sophisticated mechanisms to evade host immune responses within the central nervous system (CNS), enabling viral replication, persistence, latency, and neuropathogenesis while minimizing irreversible neuronal damage. Unlike peripheral tissues, the CNS requires tightly regulated antiviral immunity to balance effective pathogen control with the preservation of neural function. This review examines the diverse yet convergent immune evasion strategies employed by major neurotropic RNA and DNA viruses, including herpes simplex virus (HSV), varicella-zoster virus (VZV), cytomegalovirus (CMV), rabies virus (RABV), flaviviruses, alphaviruses, enteroviruses, and JC virus (JCV). We discuss viral interference with innate immune sensing pathways, including RIG-I-like receptors (RLRs) and cyclic GMP–AMP synthase–stimulator of interferon genes (cGAS–STING) signaling, inhibition of type I interferon induction and Janus kinase–signal transducer and activator of transcription (JAK–STAT) signaling, modulation of interferon-stimulated effector mechanisms, and disruption of antigen presentation and adaptive immune surveillance. The review further highlights the distinct roles of viral latency, long-term persistence, neuronal–glial interactions, and metabolic reprogramming in facilitating prolonged infection within the CNS. Emerging evidence indicates that successful neurotropic viruses rarely achieve immune evasion through complete suppression of host defenses; instead, they fine-tune antiviral responses to preserve host cell viability while preventing viral clearance. Finally, we discuss current knowledge gaps and emphasize the need for advanced human-relevant models, single-cell and spatial multi-omics, and systems-level approaches to better define virus–host interactions within the CNS. A deeper understanding of these integrated immune evasion networks may reveal novel therapeutic strategies that enhance antiviral immunity while limiting neuroinflammation and preserving neurological function.

## 1. Introduction

Neurotropic viruses comprise a diverse group of RNA and DNA viruses capable of infecting cells of the CNS, including neurons, astrocytes, microglia, and oligodendrocytes, and, depending on the viral species, establishing long-term persistent or well-defined latent infections within the central or peripheral nervous system [1,2,3,4]. Clinically important members include HSV, VZV, CMV, RABV, West Nile virus (WNV), Japanese encephalitis virus (JEV), Zika virus (ZIKV), tick-borne encephalitis virus (TBEV), poliovirus, enterovirus 71 (EV71), measles virus (MeV), Nipah virus (NiV), and JC virus [2,3,4]. Infection with these pathogens can result in outcomes ranging from acute encephalitis and chronic neuroinflammation to demyelinating disease and, for some herpesviruses, lifelong latent infection with periodic reactivation [1,2,3,4].

Although the CNS has historically been regarded as an immune-privileged site because of its restricted immune cell infiltration and tight regulation of inflammation, it is far from immunologically inactive [5,6,7]. Neurons and glial cells express pattern recognition receptors (PRRs), interferon receptors, and numerous interferon-stimulated genes (ISGs) that together form a robust intrinsic antiviral defense system [8,9,10]. However, antiviral immunity in the CNS is fundamentally shaped by the need to preserve neuronal viability and maintain neural circuit integrity [5,11]. Unlike many peripheral tissues, where inflammation-associated damage can often be repaired or tolerated, neuronal loss is frequently irreversible [6,7]. Consequently, antiviral responses in the CNS must remain tightly controlled to limit immunopathology.

This constrained immune environment has driven the evolution of sophisticated viral immune evasion strategies. Compared with viruses that are confined to peripheral tissues, neurotropic viruses often employ mechanisms that are highly cell-type-specific, persistent, and finely tuned to avoid triggering excessive immune activation [2,3,4]. While many of these strategies have been characterized in individual viruses, a broader understanding of how neurotropic pathogens collectively circumvent CNS antiviral defenses across different neural cell populations remains incomplete [4].

This review examines the molecular and cellular mechanisms by which neurotropic viruses evade antiviral immunity within the CNS, with particular emphasis on innate sensing pathways, interferon signaling, adaptive immune escape, and the unique neuronal–glial environment that supports viral persistence.

## 2. Evasion of Innate Viral Recognition

### 2.1. Antagonism of RNA Sensing Pathways

Recognition of viral nucleic acids is a critical first step in CNS antiviral defense. In neurons and glial cells, cytosolic RNA sensing is primarily mediated by RLRs, including retinoic acid-inducible gene I (RIG-I) and melanoma differentiation-associated protein 5 (MDA5) [3,12,13]. Upon binding viral RNA, these receptors undergo conformational changes and K63-linked ubiquitination, enabling interaction with the mitochondrial antiviral-signaling (MAVS). This activates TANK-binding kinase 1 (TBK1) and IκB kinase epsilon (IKKε), leading to interferon regulatory factor (IRF) 3/7 and nuclear factor kappa B (NF-κB) signaling and subsequent type I interferon production [12,14]. These signaling events have been extensively characterized through biochemical reconstitution assays, imaging approaches, and protein–protein interaction studies [14,15].

Because these early sensing events are essential for antiviral defense, they represent major targets for viral antagonism. A well-characterized example is the influenza A virus (IAV) nonstructural protein 1 (NS1), which binds viral RNA and inhibits tripartite motif-containing protein 25 (TRIM25)-mediated RIG-I ubiquitination, thereby suppressing downstream signaling and interferon induction [16,17]. Although IAV is not classified as a classical neurotropic virus, this mechanism is included as a comparative example of viral innate immune antagonism and should not be interpreted as evidence of neurotropism. Such immune evasion mechanisms may be relevant to the host response during occasional neurological complications associated with IAV infection.

Several neurotropic paramyxoviruses, including MeV and NiV, disrupt the same pathway through their V proteins, which bind MDA5 and inhibit its adenosine triphosphate (ATPase) activity, preventing filament assembly and downstream signaling. Structural analyses, co-immunoprecipitation experiments, and mutagenesis studies have confirmed these interactions and their suppressive effects on interferon responses [15,18,19]. In addition, V proteins can interfere with TRIM25-mediated RIG-I activation, illustrating convergent targeting of key regulatory checkpoints [15].

Rabies virus employs a related strategy through its phosphoprotein, which suppresses RIG-I signaling by inhibiting IRF3 phosphorylation and reducing interferon beta (IFN-β) transcription in infected neurons [20,21]. These effects have been validated using reporter assays, kinase activity measurements, and neuronal infection models.

Picornaviruses such as poliovirus and EV71 instead rely on protease-mediated disruption of signaling components. Picornaviral 2A (2A) and picornaviral 3C (3C) proteases cleave MAVS, TRIF, IRF7, and other adaptor proteins, effectively uncoupling RNA sensing from interferon induction. These proteolytic events have been characterized through biochemical in vitro protease assays, site-directed mutagenesis, and infection-based signaling studies, confirming their critical role in suppressing antiviral responses [22,23,24].

These immune evasion strategies may be especially advantageous in neurons, which often display lower constitutive expression of innate immune mediators and a reduced capacity to amplify interferon responses compared with professional immune cells [8,11]. Transcriptomic profiling, single-cell analyses, and comparative studies of CNS cell populations support this concept.

Collectively, these findings demonstrate that neurotropic viruses converge on a limited number of critical RNA sensing pathways to evade detection. Within the tightly regulated immune environment of the CNS, disruption of these early signaling events provides a substantial advantage for viral replication, persistence, and neuropathogenesis.

### 2.2. Subversion of DNA Sensing Mechanisms

In addition to RNA-sensing pathways, neurotropic DNA viruses have evolved sophisticated mechanisms to evade host DNA surveillance. Central to this defense is the cyclic GMP–AMP synthase (cGAS)–stimulator of interferon genes (STING) pathway, together with nuclear DNA sensors such as interferon gamma-inducible protein 16 (IFI16). Recognition of cytosolic double-stranded DNA by cGAS induces the synthesis of cyclic GMP–AMP (cGAMP), which activates STING on the endoplasmic reticulum membrane. Activated STING subsequently translocates to the Golgi apparatus, recruits TANK-binding kinase 1 (TBK1), and promotes IRF3 activation, culminating in type I interferon and ISG expression. The essential role of this pathway in antiviral defense has been established through CRISPR/Cas9-mediated knockout models and IFN-β reporter assays showing impaired antiviral responses in the absence of cGAS or STING [25,26,27,28].

Herpesviruses disrupt this pathway at multiple levels. HSV-1 encodes the tegument protein UL37, which deamidates cGAS, thereby impairing its enzymatic activity and reducing cGAMP production. Biochemical, structural, and functional studies, including site-directed mutagenesis, mass spectrometry, and enzymatic activity assays, have confirmed this post-translational modification and its functional consequences on antiviral signaling. Consistent with these findings, UL37-deficient viruses induce stronger type I interferon responses and exhibit attenuated replication [29].

HSV-1 further suppresses nuclear DNA sensing through the immediate-early protein ICP0, an E3 ubiquitin ligase that promotes proteasomal degradation of IFI16 and related sensing components. Co-immunoprecipitation, ubiquitination assays, and proteasome inhibition studies demonstrate direct ICP0-mediated targeting of IFI16, while immunofluorescence microscopy confirms depletion of nuclear IFI16 during infection. ICP0-deficient viruses exhibit impaired IFI16 degradation and enhanced interferon signaling [30,31].

Human cytomegalovirus (HCMV) similarly encodes several proteins that suppress DNA sensing and downstream interferon responses. The tegument protein pp65 (UL83) inhibits IRF3 activation and nuclear translocation, whereas pp71 (UL82) alters intrinsic antiviral defenses and chromatin dynamics. Proteomic analyses, chromatin immunoprecipitation assays, and protein interaction studies have identified multiple interactions between CMV proteins and components of the STING pathway [32,33,34]. Evidence from primary cell systems and in vivo models further indicates that these mechanisms contribute substantially to viral replication, persistence, and immune evasion.

Collectively, neurotropic herpesviruses employ complementary strategies that target both cytosolic and nuclear DNA-sensing pathways. By impairing cGAS activity, degrading DNA sensors, and suppressing downstream signaling intermediates, these viruses effectively attenuate antiviral responses within the CNS, thereby facilitating long-term viral persistence, and, in herpesviruses, the establishment and maintenance of latency with periodic reactivation.

To facilitate comparison across viral families, the principal mechanisms by which neurotropic viruses antagonize host nucleic acid-sensing pathways are summarized in Table 1.

## 3. Disruption of Interferon Signaling

### 3.1. Inhibition of Interferon Induction

Following PRR activation, the induction of type I interferons depends on the coordinated activation of IRF3, IRF7, and NF-κB downstream of TBK1 and IKKε signaling. Phosphorylation, dimerization, and nuclear translocation of these transcription factors drive the expression of IFN-β1 and numerous interferon-stimulated antiviral genes. Genetic and pharmacological studies, including CRISPR/Cas9-mediated gene disruption and selective kinase inhibition experiments, have confirmed the essential role of TBK1 and IRF3 in this process [35,36,37]. However, accumulating evidence indicates that the contribution of individual signaling components is highly context-dependent, varying according to cell type, viral tropism, and the kinetics of infection. In particular, neurons often exhibit delayed or attenuated type I IFN responses compared with peripheral immune cells, suggesting that canonical antiviral signaling pathways are differentially regulated within the CNS.

Many neurotropic viruses interfere directly with these signaling events. HSV-1 ICP0 not only promotes the degradation of IFI16 but also disrupts IRF3 signaling more broadly [30,38]. In parallel, the HSV-1 serine/threonine kinase US3 inhibits IRF3 phosphorylation, thereby preventing its activation and nuclear accumulation. Kinase assays and phospho-specific immunoblot analyses have demonstrated reduced IRF3 phosphorylation in infected cells expressing US3 [39]. The functional redundancy between ICP0 and US3 highlights a recurrent feature of herpesvirus immune evasion, whereby multiple viral proteins target different nodes within the same signaling pathway. Such redundancy likely increases the robustness of immune suppression and reduces dependence on a single antagonistic mechanism. Nevertheless, the relative contribution of individual viral antagonists during natural CNS infection is not completely understood, as most mechanistic studies have been performed in immortalized cell lines rather than primary neural cells or in vivo models. This represents an important limitation when extrapolating these findings to the complex cellular environment of the infected CNS.

Alphaviruses, including Sindbis virus (SINV) and Venezuelan equine encephalitis virus (VEEV), also target the TBK1–IKKε complex through their nonstructural protein 2 (nsP2), thereby suppressing IRF3 activation and IFN-β transcription. Reporter assays, protein–protein interaction studies, and mutational analyses have confirmed that nsP2-mediated disruption of kinase complex assembly contributes significantly to interferon antagonism and enhances viral replication [40,41,42]. However, the pleiotropic nature of nsP2 complicates the interpretation of these findings, as this protein regulates multiple aspects of the viral life cycle, including RNA replication, host transcriptional shutoff, and viral pathogenesis. Consequently, distinguishing its direct immunomodulatory functions from indirect effects on viral fitness has yet to be fully resolved. Moreover, although TBK1-IKKε antagonism appears to be conserved among several alphaviruses, the extent to which this mechanism determines neuroinvasion and neurovirulence in vivo is likely influenced by viral strain, tissue tropism, and the cellular composition of the infected CNS.

By suppressing interferon induction at an early stage, neurotropic viruses reduce paracrine signaling to neighboring cells and limit the establishment of a broader antiviral state within the CNS. Studies using primary neuronal cultures and experimental animal models have demonstrated that impaired interferon signaling correlates with enhanced viral replication and increased neurovirulence [43,44,45]. Nevertheless, the relationship between interferon signaling and neurovirulence is considerably more complex than a simple inverse correlation. While insufficient interferon responses facilitate viral dissemination, excessive or sustained interferon signaling within the CNS may itself contribute to synaptic dysfunction, neuronal injury, and chronic neuroinflammation. Thus, disease outcome reflects a dynamic balance between antiviral protection and immune-mediated pathology rather than the magnitude of interferon production alone.

The outcome of infection is determined not only by the magnitude of interferon production but also by the temporal and spatial regulation of antiviral signaling. Defining this balance remains a major challenge, in particular because most experimental models do not fully reproduce the cellular diversity, regional specialization and immune landscape of the human CNS.

### 3.2. Antagonism of Interferon Signaling and JAK-STAT Pathways

Even when interferons are successfully produced, many neurotropic viruses suppress downstream signaling through the JAK–STAT pathway. Binding of type I interferons to the interferon alpha/beta receptor (IFNAR) activates JAK1 and tyrosine kinase 2 (TYK2), leading to the phosphorylation of STAT1 and STAT2. Together with IRF9, these proteins form the interferon-stimulated gene factor 3 (ISGF3) complex, which translocates to the nucleus and drives ISG transcription. The importance of this pathway has been established using STAT-deficient models and pharmacological inhibition studies showing impaired antiviral defense in the absence of JAK–STAT signaling [46,47,48]. However, accumulating evidence indicates that the magnitude and composition of ISG responses differ substantially among CNS-resident cell types, suggesting that activation of the JAK–STAT pathway alone is not always predictive of antiviral efficacy. Instead, the functional outcome appears to depend on the cellular context, the timing of signaling, and the repertoire of ISGs induced within individual neural cell populations.

Although type I interferons are central to antiviral defense in the CNS, type III interferons (IFN-λs) provide an additional layer of protection, particularly at barrier interfaces. IFN-λs signal through the IFNLR1/IL-10R2 receptor complex and activate many of the same ISGs as type I IFNs, but their more restricted receptor distribution allows a more localized antiviral response. At the blood–brain barrier, IFN-λ signaling contributes to antiviral protection and barrier integrity, limiting viral neuroinvasion. Thus, the modulation of IFN-λ signaling may represent an additional mechanism of immune evasion during neurotropic viral infection [44].

Flaviviruses, including ZIKV, WNV, JEV, and TBEV, commonly target STAT signaling. ZIKV NS5 binds STAT2 and promotes its proteasomal degradation, thereby preventing ISGF3 formation and rendering infected cells resistant to interferon signaling. Co-immunoprecipitation, ubiquitination assays, proteasome inhibition studies, and mutational analyses collectively validate this mechanism [49,50,51,52]. Nevertheless, the extent to which STAT2 degradation alone accounts for viral immune evasion remains uncertain. Flaviviral NS5 proteins are multifunctional and contribute not only to immune antagonism but also to viral RNA replication and interactions with numerous host factors. Moreover, the mechanisms of STAT antagonism are not fully conserved across flaviviruses, indicating that disruption of the JAK–STAT pathway has evolved through distinct virus-specific adaptations rather than a universal strategy. Clarifying how these differences influence viral tropism, persistence, and neuropathogenesis in the CNS will require comparative studies in physiologically relevant neural models, as findings derived from transformed cell lines may not fully reflect the complexity of antiviral signaling in vivo.

NiV and Hendra virus (HeV) provide examples of henipavirus-mediated interference with interferon signaling. The V proteins of both viruses bind STAT1 and STAT2 and sequester them in cytoplasmic, high-molecular-weight complexes, thereby preventing IFN-induced nuclear accumulation and downstream antiviral signaling [53,54]. In contrast, Eastern equine encephalitis virus (EEEV) uses its capsid protein to inhibit host cell gene expression and thereby counteract the antiviral effects of IFN [55]. Western equine encephalitis virus (WEEV) capsid interferes with IRF-3-mediated, cell-intrinsic antiviral responses in neurons, including responses that are partly independent of IFN signaling [56]. Together, these examples show that neurotropic viruses can interfere with antiviral defenses at distinct levels, ranging from STAT-dependent IFN signaling to broader transcriptional and neuronal innate immune responses.

Other flaviviruses, including WNV and JEV, inhibit STAT1 phosphorylation, reducing ISG induction and enhancing viral replication in neuronal cultures [50,51,52]. Alphaviruses similarly antagonize JAK–STAT signaling through nsP2, which inhibits STAT1 phosphorylation and prevents nuclear translocation. Immunofluorescence microscopy and reporter assays demonstrate cytoplasmic retention of STAT1 and reduced ISG promoter activity in infected cells [57,58]. However, because nsP2 also regulates viral RNA synthesis, host transcriptional shutoff, and cytopathogenicity, it is unlikely that JAK–STAT antagonism alone accounts for the contribution of nsP2 to neurovirulence. Rather, its pathogenic effects probably arise from the coordinated modulation of multiple viral and host processes that collectively promote immune evasion, efficient replication and neuronal infection.

Rabies virus adopts a complementary strategy through its phosphoprotein, which binds phosphorylated STAT1 and STAT2 and sequesters them in the cytoplasm. Co-immunoprecipitation studies, confocal microscopy, and functional reporter assays confirm that this interaction prevents ISG induction and contributes to neurovirulence [20,59]. Notably, the rabies virus P protein shows that immune evasion can be achieved without the degradation of signaling molecules, emphasizing that disruption of intracellular trafficking represents an equally effective means of suppressing antiviral responses. Whether this strategy provides a selective advantage by minimizing host cell perturbation and prolonging neuronal survival remains an unresolved issue.

Despite mechanistic differences, these viruses converge on the same outcome: suppression of interferon-mediated antiviral signaling. Table 2 summarizes the principal viral antagonists, their molecular targets, and the functional consequences for interferon-mediated antiviral responses. In the CNS, where interferon responses must balance effective viral control with the preservation of neural tissue, disruption of these pathways provides a highly effective mechanism for viral survival and spread.

However, suppression of JAK–STAT signaling alone is unlikely to account for successful CNS persistence. Rather, its effectiveness depends on coordinated interactions with additional viral strategies, including the inhibition of innate immune sensing, modulation of antigen presentation, and regulation of cell survival pathways. Moreover, because interferon signaling contributes not only to antiviral defense but also to immune homeostasis within the CNS, viral interference with this pathway may have consequences that extend beyond enhanced replication, potentially influencing neuroinflammation, neuronal dysfunction, and long-term neurological sequelae. Elucidating these broader effects is an important objective for future studies.

## 4. Modulation of Interferon-Stimulated Effector Mechanisms

Beyond blocking interferon production and signaling, neurotropic viruses also interfere with downstream ISG effector pathways that directly restrict viral replication. Interference with these downstream antiviral mechanisms provides an additional layer of immune evasion, ensuring that residual interferon responses that escape upstream inhibition remain functionally ineffective. This multilayered strategy underscores the evolutionary importance of targeting multiple components of the antiviral network rather than relying on the disruption of a single signaling pathway.

One major antiviral effector is protein kinase R (PKR), which becomes activated upon binding viral double-stranded RNA. Activated PKR phosphorylates eukaryotic translation initiation factor 2 alpha (eIF2α), resulting in translational arrest and the suppression of viral protein synthesis. Western blot analyses using phospho-specific antibodies have confirmed increased eIF2α phosphorylation during infection. HSV-1 counters this response through the infected cell protein 34.5 (ICP34.5), which recruits protein phosphatase 1 (PP1) to dephosphorylate eIF2α and restore translation. Co-immunoprecipitation and functional assays demonstrate both the ICP34.5–PP1 interaction and the recovery of host translational activity [60,61,62]. Notably, ICP34.5 exemplifies how neurotropic viruses selectively manipulate host stress responses rather than abolishing them entirely. By restoring translation, HSV-1 not only promotes viral protein synthesis but also limits prolonged translational arrest that could otherwise trigger apoptosis or irreversible cellular dysfunction. This highlights the broader principle that immune evasion in the CNS is closely linked to the preservation of host cell viability, a prerequisite for successful viral persistence. The relative importance of PKR antagonism compared with the other functions of ICP34.5, including the modulation of autophagy and neurovirulence, is not completely resolved, particularly in the context of natural CNS infection.

Another important antiviral pathway is the 2′–5′-oligoadenylate synthetase (OAS)– Ribonuclease L (RNase L) system. Viral RNA sensing activates OAS enzymes, leading to the synthesis of 2′–5′ oligoadenylates that stimulate RNase L–mediated degradation of viral and cellular RNA. RNA integrity assays and quantitative reverse transcription PCR (qRT-PCR) confirm extensive RNA degradation following RNase L activation. Viruses evade this pathway by generating structured RNA decoys or expressing phosphodiesterases capable of degrading 2′-5′-oligoadenylate (2-5A) molecules, thereby preventing RNase L activation [63,64,65]. Although these mechanisms effectively preserve viral RNA integrity, their contribution to CNS infection is likely to extend beyond protection from RNA degradation. Because RNase L activation also amplifies innate immune signaling and influences inflammatory responses, its inhibition may simultaneously dampen antiviral immunity and modify the inflammatory environment within neural tissues. However, the relative importance of RNase L antagonism during neurotropic virus infection is not sufficiently characterized, as most studies have focused on peripheral infection models. Little is known about how this pathway operates in distinct CNS-resident cell populations.

The apolipoprotein B mRNA-editing enzyme catalytic polypeptide-like 3 (APOBEC3) family provides an additional layer of intrinsic antiviral defense by inducing cytidine deamination and hypermutation in viral genomes. Although originally characterized in retroviral restriction, APOBEC3 proteins also inhibit herpesviruses and other DNA viruses associated with neurotropic infection. Next-generation sequencing studies reveal characteristic APOBEC3-associated mutation signatures, while CRISPR-Cas9 and small interfering RNA (siRNA)-based perturbation experiments confirm antiviral activity [66,67,68]. However, the biological consequences of APOBEC3-mediated editing remain not completely understood. While extensive hypermutation can reduce viral fitness, sublethal editing may also increase viral genetic diversity, potentially facilitating adaptation under selective immune pressure. Thus, APOBEC3 activity may represent a double-edged sword, simultaneously restricting viral replication while potentially contributing to viral evolution. In response, DNA viruses have evolved diverse strategies to limit APOBEC3-mediated restriction by altering protein stability, intracellular localization, or access to viral nucleic acids. The diversity of these countermeasures further underscores the strong evolutionary pressure exerted by APOBEC3 proteins and suggests that evasion of intrinsic restriction factors has been a recurrent driver of host–virus co-evolution.

Importantly, neurotropic viruses often modulate rather than completely abolish ISG activity. This selective suppression may preserve sufficient host cell function to support viral persistence, particularly in nonrenewable cells such as neurons. Reporter assays using interferon-stimulated response elements (ISREs), together with CRISPR-Cas9 and RNA interference approaches, demonstrate that many viruses selectively attenuate rather than fully abolish antiviral signaling [69,70,71]. This observation supports the emerging concept that successful CNS infection depends on immune modulation rather than complete immune suppression. Excessive inhibition of host defenses may compromise essential cellular processes or trigger alternative stress pathways, whereas partial suppression allows viruses to evade immune clearance while maintaining infected neuronal viability. The molecular mechanisms that determine the threshold between protective antiviral signaling and pathological immune activation remain poorly defined. Addressing this question will require greater integration of single-cell transcriptomics, spatial profiling, and physiologically relevant CNS models to capture the cell-specific dynamics of ISG regulation during infection.

A comparative overview of these strategies, including the modulation of PKR, OAS–RNase L, APOBEC3, and ISG activity, is presented in Table 3.

**Figure 1:**
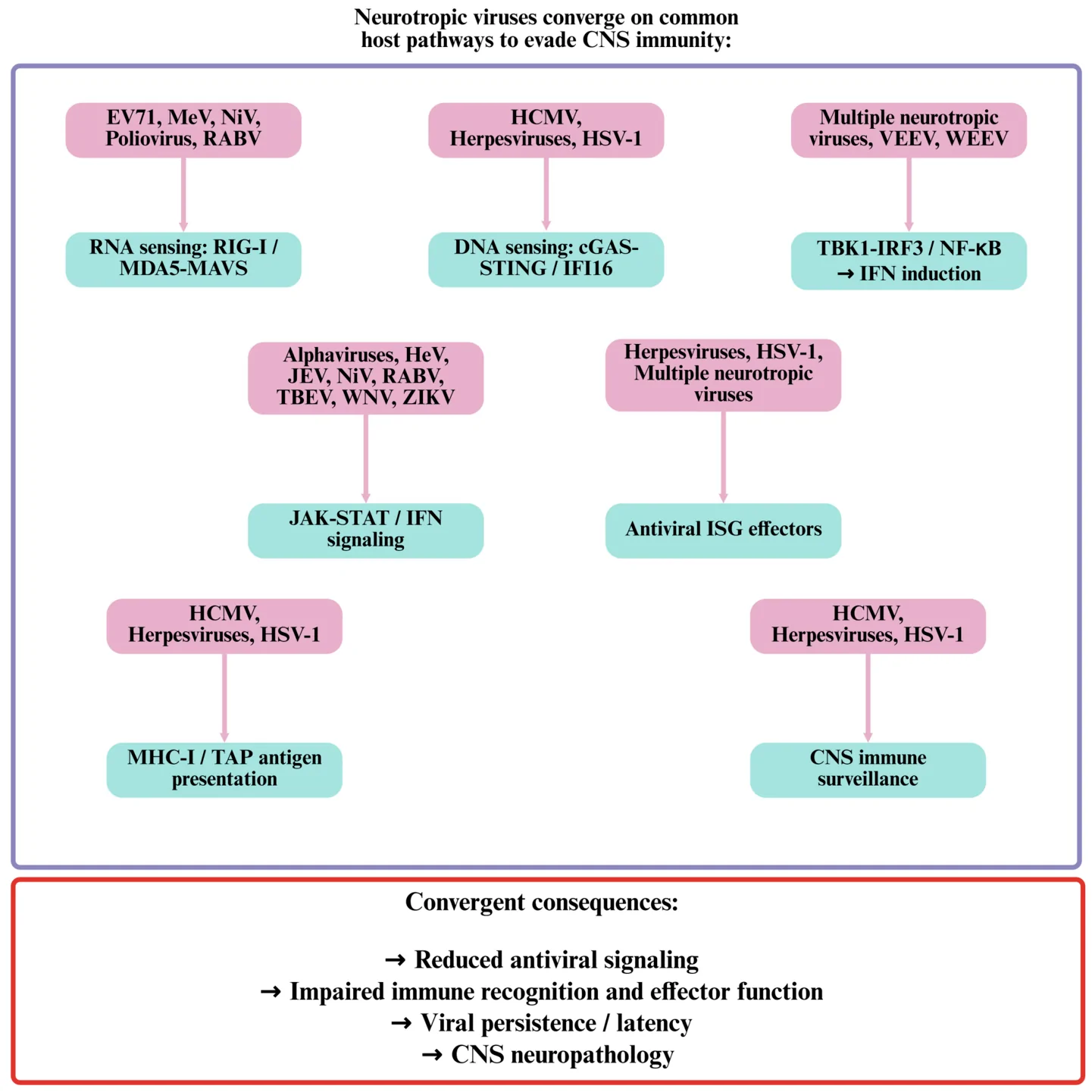
Convergent immune-evasion strategies employed by neurotropic viruses in the CNS. Diverse neurotropic viruses target shared innate and adaptive immune pathways, reducing antiviral signaling and immune recognition, and promoting long-term infection (persistence or latency, depending on the virus) and CNS neuropathology. Created in BioRender. Petrakis, V. (2026) https://BioRender.com/frsn0x3.

Despite differences among viral families, neurotropic viruses repeatedly target a limited number of antiviral pathways, as presented in Figure 1. Importantly, these pathways are interconnected, allowing the inhibition of one component to influence several downstream immune functions. This convergence highlights common evolutionary constraints shaping immune evasion within the CNS.

## 5. Evasion of Adaptive Immunity and Viral Persistence in the CNS

In addition to suppressing innate immunity, neurotropic viruses have evolved mechanisms to evade adaptive immune surveillance, enabling long-term persistence within the CNS. For clarity, latency, persistence and chronic infection are distinguished in this review. Latency refers to the maintenance of the viral genome with highly restricted viral gene expression and without sustained production of infectious virus, as exemplified by HSV-1 and VZV. Persistence is used more broadly to describe the long-term maintenance of viral material or infection despite immune pressure and may include low-level or intermittent replication. Chronic infection refers to prolonged infection characterized by sustained viral replication or antigen production and persistent host responses. These distinctions are particularly relevant in the CNS, where different viruses and cellular compartments can support distinct forms of long-term viral infection.

A principal immune evasion strategy involves the disruption of major histocompatibility complex class I (MHC I) antigen presentation, thereby limiting CD8^+^ T-cell recognition and the elimination of infected cells. This strategy is particularly advantageous in the CNS, where neurons constitutively express relatively low basal levels of MHC I and possess a limited capacity for antigen presentation compared with professional antigen-presenting cells. Rather than creating an entirely immune-invisible environment, these intrinsic characteristics raise the threshold for effective T-cell surveillance, providing neurotropic viruses with an opportunity to further weaken adaptive immune recognition.

HSV-1 and HCMV encode multiple immunoevasins that interfere with antigen processing and presentation. HSV-1 ICP47 inhibits the transporter associated with antigen processing (TAP), preventing peptide loading onto MHC I molecules. Peptide transport assays, structural analyses, and T-cell activation studies demonstrate direct ICP47-mediated suppression of antigen presentation [72,73,74]. HCMV proteins US2, US3, US6, and US11 further disrupt MHC I assembly, trafficking, and stability, as confirmed by flow cytometry and immunoprecipitation analyses [75,76,77]. The presence of multiple viral proteins targeting different stages of the same pathway suggests that efficient inhibition of antigen presentation requires a robust and redundant strategy rather than reliance on a single immunoevasin. Nevertheless, the relative contribution of these individual proteins during natural CNS infection remains incompletely understood. Most mechanistic studies have been conducted in fibroblasts or other permissive cell lines that do not completely reflect the antigen-processing machinery of neurons and glial cells. Moreover, accumulating evidence indicates that antigen presentation within the CNS is highly dynamic and is regulated by neural cell type, local inflammatory status and the stage of infection [78,79]. Consequently, the effectiveness of viral immune evasion mechanisms is likely to vary across distinct CNS microenvironments rather than presenting a uniform process throughout the CNS.

Beyond antigen presentation, some neurotropic viruses, in particular herpesviruses, establish well-defined latent infections that minimize immune recognition while preserving the capacity for future reactivation. HSV-1 and VZV establish latent infections in sensory and autonomic neurons, maintaining their genomes with highly restricted viral gene expression. During HSV-1 latency, latency-associated transcripts (LATs) contribute to the maintenance of the latent state by suppressing lytic gene expression, inhibiting apoptosis, and promoting the formation of repressive chromatin at viral lytic promoters, as evidenced by in situ hybridization, RNA sequencing, and chromatin immunoprecipitation studies [80,81,82]. Importantly, latency should not be viewed as a completely quiescent state but rather as a dynamic equilibrium between viral gene silencing and continuous immune surveillance. The limited viral transcription that persists during latency suggests that the virus remains metabolically active, requiring ongoing regulation to prevent reactivation while avoiding immune detection. Furthermore, accumulating evidence indicates that resident memory CD8^+^ T cells actively survey latently infected ganglia and contribute to maintaining viral quiescence through predominantly non-cytolytic mechanisms, including the secretion of IFN-γ and other antiviral mediators [83,84]. These findings support the concept that latency represents a dynamic equilibrium between viral immune evasion and persistent host immune control rather than complete escape from adaptive immunity. Defining the molecular events that disrupt this equilibrium and trigger viral reactivation remains one of the central unresolved issues in neurovirology.

HSV-1 also elicits distinct innate immune responses in neuronal and non-neuronal cells, reflecting differences in cellular antiviral signaling and viral immune evasion mechanisms [85].

VZV employs similar mechanisms, with latency confirmed in dorsal root and cranial nerve ganglia through PCR-based detection of viral DNA and transcriptomic analyses demonstrating restricted gene expression [86,87]. Reactivation, which occurs most frequently during aging or immunosuppression, results in herpes zoster and renewed viral replication. Despite these similarities, the molecular mechanisms governing VZV latency remain less defined than those of HSV-1, largely because of the lack of robust experimental models that accurately reproduce latent infection. Consequently, many aspects of VZV latency, including the regulatory networks controlling viral quiescence and reactivation, remain incompletely understood, which is an important gap in neurobiology.

Unlike herpesviruses, JC virus (JCV) establishes persistence primarily outside the CNS and gains access to the brain during states of impaired cellular immunity. Following reactivation, JCV can infect oligodendrocytes and cause progressive multifocal leukoencephalopathy (PML). Quantitative PCR, immunohistochemistry, and sequencing analyses have identified neurotropic viral variants associated with CNS infection and demyelinating disease [88,89,90]. However, viral reactivation alone is not sufficient to explain PML development, as only a small proportion of immunocompromised individuals develop neurological disease. This observation suggests that PML results from a complex interplay among viral genetic evolution, host immune dysfunction and CNS-specific factors rather than from immune suppression alone. The relative contribution of these factors and the mechanisms underlying the emergence of neurovirulent JCV variants are currently active areas of investigation.

The immune characteristics of the CNS further contribute to viral persistence. Restricted lymphocyte trafficking across the blood–brain barrier, low expression of co-stimulatory molecules, and local immunoregulatory activity by astrocytes and microglia all limit adaptive immune responses. In vivo imaging, flow cytometry, and transcriptomic profiling of infected neural tissues consistently demonstrate reduced T-cell effector activity within the CNS relative to peripheral tissues [2,6,91]. The concept of the CNS as an immunologically isolated compartment has been substantially revised over the past decade. The discovery of functional meningeal lymphatic vessels, enhanced understanding of CNS immune surveillance, and recognition of resident and infiltrating immune cell populations have demonstrated that immune responses within the CNS are highly regulated rather than intrinsically deficient. Consequently, viral persistence is better understood as the result of finely balanced host–virus interactions than of immune privilege alone.

Taken together, neurotropic viruses integrate multiple strategies—including impaired antigen presentation, viral latency in selected virus families, and exploitation of CNS immune regulation—to sustain long-term infection and promote neuropathogenesis. These mechanisms should not be viewed as independent processes but as components of an integrated immune evasion network that enables viruses to balance efficient replication with the preservation of host cell viability. A major challenge for future research will be to define how these complementary strategies are coordinated during different stages of infection and how their disruption might be exploited therapeutically to enhance viral clearance while minimizing immune-mediated neurological injury.

### Viral microRNAs as an Additional Immune-Evasion Mechanism

Viral microRNAs represent an additional mechanism of immune evasion, particularly during latent infection. HSV-1 encodes several miRNAs that regulate viral gene expression in latently infected neurons. In particular, miR-H2-3p targets the immediate-early transactivator ICP0, whereas miR-H6 regulates ICP4, thereby reducing the expression of viral proteins required for productive replication and reactivation [92,93]. Other HSV-1 miRNAs have also been implicated in the regulation of viral neurovirulence and host cell survival. By limiting viral protein expression while maintaining the latent viral genome, these miRNAs may contribute to persistence and the avoidance of immune recognition. However, the relative contribution of individual miRNAs to latency and immune evasion in natural human CNS infection remains incompletely defined.

The diverse mechanisms that enable neurotropic viruses to evade adaptive immune surveillance and establish long-term persistence within the CNS are summarized in Table 4, highlighting viral interference with antigen presentation, latency programs, and exploitation of the specialized CNS immune environment.

## 6. Neuronal–Glial Immune Interactions and Metabolic Constraints in CNS Infection

Immune evasion by neurotropic viruses occurs within a highly specialized cellular environment in which interactions between neurons and glial cells strongly influence antiviral responses. Neurons regulate glial activity through neurotransmitters, neuropeptides, and immunomodulatory ligands, whereas microglia and astrocytes serve as the principal innate immune effectors of the CNS. Co-culture systems, in vivo imaging, and single-cell transcriptomic studies demonstrate that these interactions coordinate antiviral defense while limiting inflammatory damage [2,6,91]. Neurons also possess substantial cell-intrinsic antiviral defenses that operate independently of professional immune cells. These include nucleic acid sensing and interferon-dependent pathways that can restrict viral replication while limiting excessive inflammatory signaling. The balance between intrinsic neuronal immunity and signals received from surrounding glial cells is therefore an important determinant of the outcome of CNS infection [94]. Importantly, antiviral immunity within the CNS should not be viewed as the sum of independent cellular responses, but rather as an emergent property of a highly interconnected cellular network. Consequently, viral manipulation of neuron–glia communication may profoundly alter immune function without directly targeting canonical antiviral signaling pathways. This concept has broadened the traditional view of viral immune evasion, emphasizing that the disruption of intercellular communication can be as biologically significant as the inhibition of intracellular antiviral signaling.

A major mechanism of neuronal–microglial communication involves the C-X3-C motif chemokine ligand 1 (CX3CL1)–C-X3-C motif chemokine ligand 1 (CX3CR1) and CD200–CD200R signaling axes. Neuronal CX3CL1 maintains microglia in a neuroprotective state through CX3CR1 signaling, while CD200 suppresses excessive activation through CD200R. Disruption of these pathways during viral infection promotes inflammatory cytokine production, oxidative stress, and neuronal injury. Genetic knockout models and immunohistochemical analyses demonstrate enhanced microglial activation and neurotoxicity when these regulatory pathways are impaired [95,96,97]. However, whether neurotropic viruses actively target these signaling systems as primary immune evasion mechanisms or whether their disruption occurs predominantly as a secondary consequence of infection-induced neuroinflammation remains unresolved. Distinguishing direct viral modulation from indirect inflammatory effects represents an important challenge, particularly because these pathways are simultaneously influenced by neuronal injury, cytokine signaling, and alterations in CNS homeostasis.

Astrocytes also contribute substantially to CNS immunity by regulating blood–brain barrier integrity, cytokine production, and metabolic homeostasis. Viral infection can alter astrocytic signaling and shift these cells toward either pro-inflammatory or immunosuppressive phenotypes. Transcriptomic profiling and cytokine analyses in infected astrocyte cultures support the context-dependent nature of these responses [98,99]. Nevertheless, the binary classification of astrocytes as either pro-inflammatory or immunosuppressive likely oversimplifies their functional diversity. Increasing evidence from single-cell transcriptomic and spatial profiling studies indicates that astrocyte activation exists along different dynamic states that vary according to brain region, disease stage, and local inflammatory conditions. Understanding how neurotropic viruses influence this cellular heterogeneity and whether specific astrocyte states preferentially promote viral persistence or neuropathology is an important area for future investigation. Microglia are also important mediators of antiviral surveillance in the CNS, although excessive or prolonged activation can contribute to neuroinflammation and neuronal injury [100].

Oligodendrocytes represent another important cellular compartment in CNS infection, particularly because of their role in maintaining myelin and their vulnerability to specific neurotropic viruses. Oligodendrocytes exhibit distinct intrinsic antiviral responses, including relatively limited type I IFN induction and responsiveness compared with microglia, which may influence their susceptibility to viral infection [101]. This is particularly evident in JCV infection, where productive infection of oligodendrocytes is a central feature of PML and demyelination [102]. Compared with neurons, astrocytes, and microglia, however, the cell-intrinsic antiviral responses of oligodendrocytes and their interactions with neighboring immune and glial cells remain less well characterized. Defining these cell-specific responses may help clarify why certain viruses preferentially target oligodendrocytes and how infection contributes to demyelination and CNS injury.

The cellular heterogeneity of the infected CNS presents an important challenge for understanding how neurotropic viruses interact with distinct neuronal and glial populations. Conventional bulk transcriptomic and proteomic approaches can obscure cell-type-specific responses by averaging signals across heterogeneous populations. Single-cell RNA sequencing and related single-cell multi-omic approaches provide an opportunity to resolve antiviral responses at the level of individual cell populations and to distinguish cell-specific programs of viral sensing, interferon signaling, antigen presentation, metabolic remodeling, and inflammatory activation. Such approaches could help determine whether viral immune evasion mechanisms are preferentially associated with particular neuronal, microglial, or astrocytic states and could identify cellular populations that serve as reservoirs or sites of persistent infection [103,104,105].

Spatial transcriptomic and spatial proteomic approaches provide an additional dimension by preserving the anatomical context of these cell-specific responses. This is particularly relevant in the CNS, where the functional consequences of viral infection are strongly influenced by the proximity of infected neurons to microglia, astrocytes, infiltrating immune cells, and vascular structures. Integrating spatial information with single-cell profiles could therefore reveal localized immune niches in which antiviral signaling, inflammatory responses, and viral persistence converge. Such approaches may also help distinguish protective immune activation from pathological neuroinflammation by identifying the cellular interactions and spatially restricted signaling programs associated with neuronal survival or injury [103,106,107,108].

The integration of single-cell transcriptomics, spatial profiling, epigenomic, proteomic, and viral RNA measurements could further provide a systems-level view of neurotropic viral immune evasion. Rather than considering viral antagonists and host pathways as isolated interactions, multi-omic analyses could identify coordinated cell-state transitions and regulatory networks associated with viral persistence, interferon resistance, or inflammatory damage. These datasets may also facilitate the identification of therapeutic targets that are selectively active in pathogenic cell states while preserving antiviral functions in neighboring cells. However, careful validation will be required because transcript abundance does not necessarily reflect protein activity or pathway function, and the temporal dynamics of infection may be difficult to reconstruct from static tissue samples. Combining longitudinal experimental models with spatial and single-cell multi-omics may therefore be particularly valuable for defining how CNS immune states evolve from early antiviral responses to persistent infection and neuroinflammation [109,110].

In addition to cellular signaling, metabolic regulation has emerged as a critical determinant of antiviral immunity in the CNS. Activation of microglia and astrocytes is accompanied by metabolic reprogramming characterized by enhanced glycolysis and altered mitochondrial activity. Extracellular flux assays, isotope tracing studies, and metabolic flux analyses demonstrate increased glycolytic activity in activated glial cells [111,112,113]. However, these metabolic adaptations are not universally beneficial. Although they facilitate rapid immune effector functions, prolonged or excessive metabolic reprogramming may disrupt neuronal metabolic homeostasis, promote oxidative stress, and exacerbate neurodegeneration. Consequently, metabolic remodeling should be viewed not merely as a consequence of immune activation but as a critical regulator of the balance between antiviral protection and tissue injury.

Emerging evidence indicates that neurotropic viruses exploit these metabolic alterations to support replication and persistence. Metabolomic profiling and pharmacological inhibition studies suggest that viruses can manipulate host biosynthetic and energy-producing pathways to favor infection, while the disruption of these pathways can reduce viral replication and alter disease progression [114,115,116]. Nevertheless, whether these metabolic alterations primarily reflect virus-driven reprogramming or represent secondary adaptations of infected cells to inflammatory stress remains an area of active investigation. Moreover, the metabolic requirements of viruses are unlikely to be uniform across different neural cell types or stages of infection, highlighting the need for greater consideration of cell-specific metabolic heterogeneity in future studies. Advances in single-cell metabolomics and spatial metabolic imaging are expected to provide important insights into these unresolved questions.

Overall, immune evasion by neurotropic viruses extends beyond direct interference with antiviral signaling pathways. Rather than targeting individual immune mechanisms in isolation, these pathogens exploit the integrated network of immune signaling, intercellular communication, and metabolic regulation that defines the CNS microenvironment. This systems-level perspective suggests that viral persistence is achieved through the coordinated modulation of multiple host processes rather than through a single dominant immune evasion strategy. Consequently, therapeutic approaches aimed exclusively at enhancing antiviral immunity may prove insufficient unless they also consider the metabolic and cellular context in which immune responses occur. Defining how these interconnected pathways collectively influence viral persistence and neurological injury remains a major challenge and an important direction for future neurovirology research.

Table 5 provides an overview of these interconnected mechanisms and their contributions to CNS infection and neuropathogenesis.

**Figure 2:**
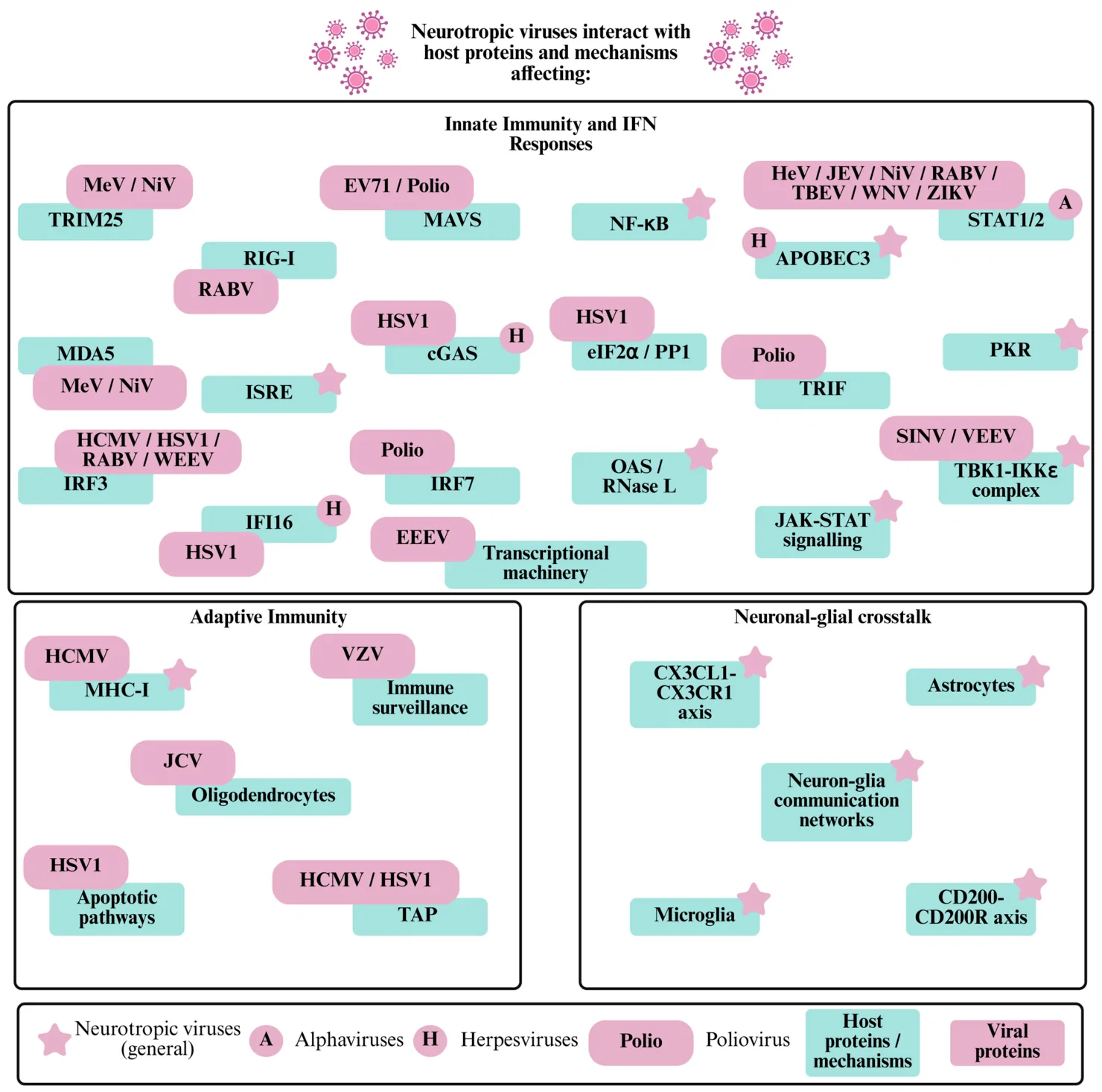
Integrated overview of viral–host immune and CNS-specific interactions targeted by neurotropic viruses. Major neurotropic RNA and DNA viruses target common host proteins and signaling pathways involved in innate immune sensing (RIG-I/MDA5, cGAS, MAVS, TRIF, TBK1–IKKε, IRF3/IRF7, NF-κB, JAK–STAT, PKR, OAS/RNase L, APOBEC3), adaptive immune responses (MHC-I antigen presentation, TAP, apoptosis, and immune surveillance), and neuronal–glial communication (CX3CL1–CX3CR1, CD200–CD200R, astrocytes, microglia, and neuron–glia signaling networks). Individual virus names indicate experimentally reported interactions with the corresponding host targets discussed in this review. (★ shared/convergent host targets; A: alphaviruses; H: herpesviruses). Created in BioRender. Petrakis, V. (2026) https://BioRender.com/hv3w7zn.

Figure 2 summarizes the dynamic viral–host immune interactions in the CNS, illustrating how distinct neurotropic viruses target shared signaling hubs involved in innate immune recognition, interferon responses, adaptive immunity, and neuron–glia communication to promote persistence and neuropathogenesis.

## 7. Therapeutic Opportunities: Restoring Antiviral Immunity While Limiting Neuroinflammation

The integrated immune evasion mechanisms described above provide a framework for identifying therapeutic strategies that target both viral persistence and the host pathways that permit sustained infection within the CNS. Persistent neurotropic viral infections represent a particular therapeutic challenge because effective viral clearance must be achieved without inducing excessive inflammation or irreversible neuronal injury. Consequently, therapeutic approaches should aim not simply to maximize antiviral immunity, but to restore an appropriate balance between viral control, immune surveillance, and the preservation of neural function [117,118]. The CNS may also serve as a specialized reservoir in which persistent viruses can remain protected from conventional immune-mediated clearance, emphasizing the need for strategies that address both viral persistence and the local immune environment [117].

One potential approach is the restoration of antiviral sensing and interferon responses that are selectively inhibited by viral antagonists. The mechanisms described in Section 2 and Section 3 identify RIG-I/MDA5, cGAS–STING, TBK1–IRF3, and JAK–STAT signaling as recurrent targets of viral immune evasion. Therapeutic interventions that restore these pathways could potentially enhance intrinsic antiviral activity and strengthen paracrine protection of neighboring CNS cells. However, indiscriminate activation of innate immune signaling may be detrimental in the CNS, where prolonged interferon responses and inflammatory activation can contribute to neuronal dysfunction and tissue injury. Therefore, therapeutic modulation of these pathways will likely require careful consideration of dose, timing, duration, and cellular specificity rather than sustained global activation of antiviral immunity. Recent work emphasizing the coordinated contribution of myeloid cells and CD8^+^ T cells to antiviral protection in the infected CNS further supports the concept that therapeutic efficacy depends on appropriately regulated local immune responses [118].

A complementary strategy is to target viral proteins and mechanisms that directly establish or maintain persistence. Viral genome targeting, RNA interference, antisense oligonucleotides, genome-editing approaches, and conventional antiviral compounds have been proposed as potential methods for reducing persistent viral reservoirs or maintaining viruses in a nonproductive state. These approaches may be particularly valuable for viruses that establish latency or long-term persistence in neurons, where complete immune-mediated elimination may be difficult to achieve. In the context of herpesvirus infection, for example, therapeutic strategies that interfere with latency or selectively reactivate and subsequently eliminate latent virus represent potential avenues for future investigation. However, the feasibility of such approaches in the human CNS remains uncertain, and strategies that induce viral reactivation would need to be combined with effective antiviral control to prevent dissemination and neuronal damage [117].

The immune evasion mechanisms discussed in Section 5 also suggest opportunities to improve adaptive immune surveillance. Restoring antigen presentation or overcoming viral inhibition of MHC-I/TAP pathways could increase the recognition of infected cells by CD8^+^ T lymphocytes. Nevertheless, the enhancement of cytotoxic immunity within the CNS must be carefully controlled because the elimination of infected neurons may itself result in irreversible neurological damage. Strategies that promote non-cytolytic antiviral functions, including local interferon-mediated control and other antiviral effector mechanisms, may therefore offer advantages over approaches that rely exclusively on the destruction of infected cells. The observation that resident memory CD8^+^ T cells can contribute to control of latent infection through predominantly non-cytolytic mechanisms further supports the potential value of immune strategies that suppress viral replication while preserving neuronal viability.

The neuron–glia interactions described in Section 6 provide an additional therapeutic target. Rather than broadly suppressing microglial or astrocytic activation, future interventions could seek to modify specific inflammatory or immunometabolic states that promote neuronal injury while preserving antiviral functions. Modulation of pathways such as CX3CL1–CX3CR1 and CD200–CD200R may help regulate excessive microglial activation, whereas interventions targeting cellular metabolism could potentially reduce inflammation-associated metabolic stress. However, the substantial heterogeneity of microglia and astrocytes across CNS regions and stages of infection means that therapeutic manipulation of these pathways will require cell- and context-specific approaches. Current evidence indicates that myeloid cells and T cells have complementary roles in antiviral protection within the CNS, further emphasizing that therapeutic modulation should preserve rather than broadly eliminate local immune surveillance [118].

An important consideration for future therapeutic development is the blood–brain barrier and the distinctive pharmacological environment of the CNS. Even when an antiviral compound is effective against a virus in peripheral tissues, therapeutic concentrations may not be achieved within infected neural cells. Recent analyses of CNS antiviral treatment have therefore emphasized the importance of drug penetration across the blood–brain and blood–cerebrospinal fluid barriers, intracellular drug distribution, and the relationship between pharmacokinetics and viral replication within different CNS compartments [119]. These considerations are particularly relevant for persistent infections, in which antiviral treatment may need to reach anatomically restricted viral reservoirs over prolonged periods.

Taken together, these observations suggest that the most effective future interventions may combine direct antiviral activity with precisely regulated host-directed immunomodulation. Rather than targeting a single immune evasion mechanism, combination approaches could simultaneously reduce viral replication or persistence, restore selected antiviral pathways, and limit the pathological activation of microglia and other inflammatory processes. Such strategies would need to be adapted to the stage of infection, the identity of the infected CNS cell, and the balance between viral burden and immune-mediated injury. The development of human-relevant organoid, co-culture, single-cell, spatial and computational models will be particularly important for identifying therapeutic windows in which antiviral immunity can be enhanced without compromising neuronal integrity. Ultimately, translating the molecular understanding of neurotropic viral immune evasion into therapy will require an integrated approach in which viral eradication, immune regulation, drug delivery, and neuroprotection are considered simultaneously [117,118,119].

## 8. Conclusions

Neurotropic viruses have evolved a diverse repertoire of immune evasion mechanisms that, despite considerable molecular variation, converge on a limited number of critical host pathways. By targeting innate immune sensing, interferon signaling, antigen presentation, and intrinsic antiviral defenses, these viruses establish an environment that favors viral persistence while minimizing irreversible damage to infected neural cells. Rather than inducing global immune suppression, they selectively modulate antiviral responses enabling efficient replication or long-term persistence without compromising the integrity of the host tissue. This capacity to fine-tune, rather than completely suppress, host immunity represents a hallmark of successful neurotropic viruses and reflects the unique immunological constraints of the CNS.

Advances in CNS immunology have fundamentally reshaped our understanding of virus–host interactions in the brain. The long-standing view of the CNS as an immunologically passive or immune-privileged organ has given way to a more dynamic model in which antiviral immunity emerges from complex interactions and communication among neurons, glial cells, infiltrating immune populations, and the local metabolic tissue microenvironment. Within this framework, viral persistence should no longer be viewed solely as a consequence of viral immune evasion but as the result of a continuously evolving dynamic equilibrium between viral adaptation and tightly regulated host immune responses. Importantly, persistent infection encompasses distinct biological states, including well-defined latency in herpesviruses and prolonged or recurrent infection in other neurotropic viruses, and these states should not be considered interchangeable. Understanding how this equilibrium is established, maintained, and ultimately disrupted during viral reactivation or neurological disease remains one of the central challenges in contemporary neurovirology.

Although considerable progress has been made in identifying individual viral immune evasion mechanisms, many fundamental questions remain unresolved. Much of our current knowledge derives from mechanistic studies of individual viruses or simplified experimental systems that only partially capture the cellular complexity and spatial organization of the human CNS. Moreover, relatively little is known about how multiple immune evasion mechanisms operate simultaneously and interact with one another within infected tissues or how their effectiveness varies across distinct neural cell types and different stages of infection. Addressing these questions will require the integration of physiologically relevant human model systems with emerging technologies, including single-cell and spatial multi-omics, high-resolution imaging, and systems-level computational approaches.

Future studies should move beyond the characterization of individual viral antagonists toward a more integrated understanding of the complex networks that coordinate antiviral immunity, immune surveillance, cellular communication, and tissue metabolic homeostasis within the CNS. Such an integrated perspective will not only deepen our understanding of the mechanisms governing neurotropic virus persistence but may also reveal new therapeutic opportunities that preserve neuronal function, prevent long-term neurological complications, and promote durable antiviral immunity while minimizing immunopathology.

## Figures and Tables

**Table 1:** Viral antagonism of innate nucleic acid sensing pathways relevant to neurotropic CNS infection.

Virus/Viral Family	Viral Protein	Host Target	Affected Pathway	Mechanism	Consequence	Ref.
IAV	NS1	TRIM25/RIG-I	RIG-I signaling	TRIM25/RIG-I ubiquitination inhibition and viral RNA binding	Interferon signaling blockade and reduced IFN induction	[16,17]
MeV	V protein	MDA5; TRIM25	RLR signaling	MDA5 binding, ATPase activity inhibition, and TRIM25-mediated RIG-I activation interference	Filament assembly blockade and interferon response suppression	[15,18,19]
NiV	V protein	MDA5; TRIM25	RLR signaling	MDA5 activation inhibition and TRIM25-mediated RIG-I signaling disruption	Downstream antiviral signaling suppression and IFN production reduction	[15,18,19]
RABV	Phosphoprotein (P)	IRF3/RIG-I pathway	RIG-I-IRF3 signaling	IRF3 phosphorylation inhibition and downstream IFN-β transcription suppression	Antiviral signaling reduction in infected neurons	[20,21]
Poliovirus	2A and 3C proteases	MAVS, TRIF, IRF7	RNA sensing and IFN induction	Proteolytic cleavage of adaptor and signaling proteins	RNA sensing–interferon induction uncoupling	[22,23]
EV71	2A protease	MAVS	MAVS signaling	MAVS cleavage and antiviral signaling complex disruption	Type I interferon response suppression	[22,24]
HSV-1	UL37	cGAS	cGAS–STING pathway	cGAS deamidation and enzymatic activity impairment	cGAMP synthesis reduction and IFN signaling suppression	[29]
	ICP0	IFI16	Nuclear DNA sensing	Ubiquitin-mediated IFI16 proteasomal degradation	IRF3 suppression and interferon induction reduction	[30,31]
HCMV	pp65 (UL83)	IRF3	STING/IRF3 signaling	IRF3 activation inhibition and nuclear translocation blockade	Interferon production reduction	[32,33,34]
	pp71 (UL82)	Intrinsic antiviral factors/Chromatin regulators	DNA sensing and intrinsic immunity	Chromatin dynamics alteration and intrinsic antiviral defense suppression	Viral persistence facilitation and immune evasion	[32,33,34]
Herpesvirus	Multiple viral proteins	cGAS, IFI16, STING pathway intermediates	Cytosolic and nuclear DNA sensing	Combined DNA sensing and downstream signaling suppression	CNS persistence, latency, and reactivation promotion	[29,30,31,32,33,34]

**Table 2:** Disruption of interferon induction and JAK–STAT signaling by neurotropic viruses.

Virus/Viral Family	Viral Protein	Host Target	Affected Pathway	Mechanism	Consequence	Ref.
HSV-1	ICP0	IRF3 signaling components	Interferon Induction	IRF3 signaling disruption and IFI16 degradation	IFN-β production reduction and antiviral signaling suppression	[30,38]
	US3 kinase	IRF3	TBK1-IRF3 pathway	IRF3 phosphorylation inhibition	IRF3 activation blockade and downstream interferon induction suppression	[39]
SINV	nsP2	TBK1-IKKε Complex	Interferon Induction	IRF3-activating kinase complex assembly disruption	IFN-β transcription suppression and viral replication enhancement	[40,41,42]
VEEV	nsP2	TBK1-IKKε Complex	Interferon Induction	TBK1/IKKε pathway disruption and IRF3 suppression	Interferon induction reduction and replication enhancement	[40,41,42]
ZIKV	NS5	STAT2	JAK–STAT signaling	STAT2 proteasomal degradation promotion	ISGF3 formation blockade and ISG transcription suppression	[49,50,51,52]
WNV	Viral antagonists (multiple)	STAT1	JAK–STAT signaling	STAT1 phosphorylation inhibition	ISG induction reduction and neuronal replication enhancement	[50,51,52]
JEV	Viral antagonists (multiple)	STAT1	JAK–STAT signaling	STAT1 phosphorylation inhibition	Interferon-mediated antiviral defense impairment	[50,51,52]
NiV	V protein	STAT1 and STAT2	Type I IFN signaling	STAT1/STAT2 binding and cytoplasmic sequestration in high-molecular-weight complexes	Prevention of STAT1/STAT2 nuclear accumulation and downstream antiviral signaling	[53]
HeV	V protein	STAT1 and STAT2	Type I IFN signaling	STAT1/STAT2 binding and cytoplasmic sequestration in high-molecular-weight complexes	Prevention of STAT1/STAT2 nuclear accumulation and downstream antiviral signaling	[54]
EEEV	Capsid protein	Host transcriptional machinery	Host cell gene expression/IFN-mediated antiviral response	Inhibition of host cell gene expression	Attenuation of IFN-mediated antiviral effects	[55]
WEEV	Capsid protein	IRF-3	IRF-3-dependent cell-intrinsic antiviral response; partly IFN-independent	Interference with IRF-3-mediated antiviral responses	Impairment of neuronal innate antiviral defenses, including IFN-independent responses	[56]
TBEV	Viral antagonists (multiple)	STAT signaling components	JAK–STAT signaling	Interferon signaling pathway disruption	Antiviral response resistance enhancement	[49,50,51,52]
Alphaviruses	nsP2	STAT1	JAK–STAT signaling	STAT1 phosphorylation inhibition and nuclear translocation blockade	STAT1 cytoplasmic retention and ISG expression reduction	[57,58]
RABV	Phosphoprotein (P)	Phosphorylated STAT1 and STAT2	JAK–STAT signaling	Phosphorylated STAT protein cytoplasmic sequestration	ISG induction suppression and neurovirulence enhancement	[20,59]
Neurotropic viruses	Multiple viral antagonists	TBK1, IRF3, NF-κB signaling	Early Interferon induction	IFN regulatory transcription factor inhibition	Paracrine antiviral signaling suppression and neurovirulence enhancement	[35,36,37,38,39,40,41,42,43,44,45]
	Multiple viral proteins	JAK–STAT pathway components	Interferon-mediated antiviral signaling	STAT activation, translocation, and ISGF3 formation inhibition	Antiviral immunity suppression and CNS viral survival/spread enhancement	[45,46,47,48,49,50,51,52,53,54,55,56,57,58,59]

**Table 3:** Modulation of interferon-stimulated effector mechanisms by neurotropic viruses.

Virus/Viral Family	Viral Protein/Strategy	Host Target	Affected Pathway	Mechanism	Consequence	Ref.
HSV-1	ICP34.5	eIF2α/PP1	PKR-eIF2α pathway	PP1 recruitment and eIF2α dephosphorylation	Translation restoration and PKR-mediated translational arrest prevention	[60,61,62]
Neurotropic Viruses	Viral dsRNA antagonism	PKR	PKR signaling	PKR activation and downstream phosphorylation inhibition	Viral protein synthesis and replication maintenance	[60,61,62]
	Structured RNA decoys	OAS/RNase L	OAS-RNase L pathway	RNase L activation inhibition via RNA structure formation	Viral and cellular RNA degradation reduction	[63,64,65]
	Viral phosphodiesterases	2-5A	OAS-RNase L pathway	2-5A signaling molecule degradation and RNase L inhibition	RNase L-mediated antiviral RNA degradation inhibition	[63,64,65]
	Partial ISG antagonism	ISRE-regulated antiviral genes	Interferon-stimulated effector responses	ISG activity modulation rather than complete suppression	Host cell viability preservation and viral persistence maintenance	[69,70,71]
	Multiple viral immune modulators	PKR, OAS/RNase L, APOBEC3 pathways	Downstream ISG effector mechanisms	Selective antiviral effector system suppression	Neuronal replication support with reduced cellular damage	[60,61,62,63,64,65,66,67,68,69,70,71]
DNA viruses associated with neurotropic infection	Multiple viral antagonists	APOBEC3 proteins	APOBEC3 restriction pathway	APOBEC3 stability and localization alteration	Cytidine deamination and viral genome hypermutation evasion	[66,67,68]
Herpesviruses	Multiple viral proteins	APOBEC3 family members	Intrinsic antiviral restriction	APOBEC3 activity and localization modulation	Antiviral mutagenesis reduction and persistence enhancement	[66,67,68]

**Table 4:** Mechanisms of adaptive immune evasion and viral persistence in the CNS.

Virus/Viral Family	Viral Protein/Strategy	Host Target	Affected Pathway	Mechanism	Consequence	Ref.
HSV-1	ICP47	TAP transporter	MHC-I antigen presentation	MHC I antigen presentation blockade via ER peptide transport inhibition	CD8^+^ T-cell recognition reduction	[72,73,74]
	LATs	Lytic viral gene expression/Apoptotic pathways	Viral latency and immune evasion	Lytic gene expression suppression and apoptosis inhibition	Antigen presentation minimization and long-term neuronal persistence	[80,81,82]
	Episomal latency	Host neuronal immune surveillance	Adaptive immune recognition	Highly restricted viral gene expression during latency	CD8^+^ T-cell detection prevention and persistence support	[80,81,82]
HCMV	US2	MHC-I molecules	Antigen presentation	MHC-I heavy chain degradation promotion	Surface MHC-I expression impairment and T-cell recognition reduction	[75,76,77]
	US3	MHC-I assembly machinery		MHC-I endoplasmic reticulum retention	MHC-I trafficking and antigen presentation inhibition	[75,76,77]
	US6	TAP transporter		TAP-mediated peptide translocation inhibition	MHC-I antigenic peptide loading suppression	[75,76,77]
	US11	MHC-I molecules		MHC I dislocation and degradation promotion	Adaptive immune evasion	[75,76,77]
VZV	Restricted latent gene expression	Host immune surveillance	Viral latency	Maintenance of a latent state with restricted viral gene expression	Latent infection with periodic reactivation	[86,87]
JCV	Neurotropic viral variants	Oligodendrocytes/Cellular immunity	CNS immune surveillance	Impaired cellular immunity exploitation for CNS infection	PML development	[88,89,90]
Herpesvirus	Latency programs	MHC-I antigen presentation and T-cell surveillance	Adaptive immunity	Restricted viral protein synthesis and transcriptionally silent genomes	Long-term latent infection with periodic reactivation	[72,73,74,75,76,77,78,79,80,81,82,83,84,85,86,87]
Neurotropic Viruses	Exploitation of CNS immune privilege	BBB, astrocytes, microglia, T-cell trafficking	CNS adaptive immune responses	Restricted lymphocyte trafficking and local immunoregulation	T-cell effector activity limitation and persistence support	[2,6,91]
	Multiple immunoevasion mechanisms	MHC-I pathway, T cells, CNS immune regulation	Adaptive immune surveillance	Impaired antigen presentation, viral latency in selected virus families, and CNS immune modulation	Long-term viral persistence and contribution to neuropathogenesis	[2,72,73,74,75,76,77,78,79,80,81,82,83,84,85,86,87,88,89,90,91]

**Table 5:** Neurotropic viral exploitation of neuronal–glial immune interactions and CNS immunometabolism.

Virus/Viral Family	Viral Strategy	Host Target	Affected Pathway	Mechanism	Consequence	Ref.
Neurotropic Viruses	Modulation of neuron-glia signaling	CX3CL1-CX3CR1 axis	Neuronal-microglial communication	Neuroprotective signaling disruption between neurons and microglia	Inflammatory cytokine production and neurotoxicity enhancement	[95,96,97]
	Disruption of inhibitory immune signaling	CD200-CD200R axis	Microglial immune regulation	Inhibitory signaling reduction and microglial activation enhancement	Oxidative stress, inflammation, and neuronal injury promotion	[95,96,97]
	Inflammation-induced neuronal signaling disruption	Neuron-glial communication networks	CNS immune homeostasis	Ligand expression alteration and inflammation-mediated signaling disruption	CNS immune regulation destabilization	[95,96,97]
	Astrocyte immune modulation	Astrocytes	Cytokine signaling and BBB regulation	Astrocyte pro-inflammatory/immunosuppressive phenotype shift	CNS immune response and BBB integrity alteration	[98,99]
	Alteration of astrocytic signaling	Astrocyte metabolic and immune pathways	CNS homeostasis	Astrocyte signaling reprogramming during infection	Inflammatory response and viral persistence modulation	[98,99]
	Induction of glial metabolic reprogramming	Microglia and astrocytes	Immunometabolic pathways	Enhanced glycolysis and altered mitochondrial activity in activated glia	Immune activation support and neuronal metabolic stress	[111,112,113]
	Exploitation of host metabolic pathways	Cellular biosynthetic and energy-producing pathways	CNS metabolism	Metabolic pathway modulation for viral replication and persistence	Infection efficiency and disease progression enhancement	[114,115,116]
	Competition for metabolic resources	Neurons versus activated glia	CNS metabolic homeostasis	Glial glycolytic shift and neuronal energy competition	Neuronal vulnerability and dysfunction enhancement	[111,112,113]
	Manipulation of immunometabolic balance	Neuron-glia metabolic interaction	CNS antiviral immunity	CNS-specific metabolic and inflammatory constraint exploitation	Persistence support and neuropathogenesis contribution	[111,112,113,114,115,116]
	Coordinated immune and metabolic exploitation	Neurons, astrocytes, microglia	Integrated CNS immune environment	Cellular signaling disruption and metabolic manipulation	Permissive environment for prolonged viral persistence and CNS injury	[2,6,91,95,96,97,98,99,111,112,113,114,115,116]

## Data Availability

No new data were created or analyzed in this study. Data sharing is not applicable to this article.

## Abbreviations

The following abbreviations are used in this manuscript:

2A	Picornaviral 2A protease
2-5A	2′-5′-oligoadenylate
3C	Picornaviral 3C protease
APOBEC3	Apolipoprotein B mRNA-editing enzyme catalytic polypeptide-like 3
ATPase	Adenosine triphosphate
BBB	Blood–brain barrier
cGAMP	Cyclic GMP-AMP
cGAS	Cyclic GMP–AMP synthase
cGAS–STING	Cyclic GMP–AMP synthase–stimulator of interferon genes signaling pathway
CD8^+^ T cells	Cluster of differentiation 8-positive T cells
CD200	Cluster of differentiation 200
CD200R	Cluster of differentiation 200 receptor
CMV	Cytomegalovirus
CNS	Central nervous system
CRISPR/Cas9	Clustered regularly interspaced short palindromic repeats/CRISPR-associated protein 9
CX3CL1	C-X3-C motif chemokine ligand 1
CX3CR1	C-X3-C motif chemokine receptor 1
EEEV	Eastern equine encephalitis virus
eIF2α	Eukaryotic translation initiation factor 2 alpha
EV71	Enterovirus 71
HCMV	Human cytomegalovirus
HeV	Hendra virus
HSV	Herpes simplex virus
HSV-1	Herpes simplex virus 1
ICP0	Infected cell protein 0
ICP34.5	Infected cell protein 34.5
ICP47	Infected cell protein 47
IAV	Influenza A virus
IFI16	Interferon gamma-inducible protein 16
IFN-β	Interferon beta
IFN-β1	Interferon beta 1
IFNAR	Interferon alpha/beta receptor
IKKε	IκB kinase epsilon
IRF3	Interferon regulatory factor 3
IRF7	Interferon regulatory factor 7
IRF9	Interferon regulatory factor 9
ISGs	Interferon-stimulated genes
ISGF3	Interferon-stimulated gene factor 3
ISREs	Interferon-stimulated response elements
JAK	Janus kinase
JAK1	Janus kinase 1
JAK–STAT	Janus kinase-signal transducer and activator of transcription signaling pathway
JCV	JC virus
JEV	Japanese encephalitis virus
LATs	Latency-associated transcripts
MAVS	Mitochondrial antiviral-signaling protein
MDA5	Melanoma differentiation-associated protein 5
MeV	Measles virus
MHC-I	Major histocompatibility complex class I
NF-κB	Nuclear factor kappa B
NiV	Nipah virus
NS1	Nonstructural protein 1
nsP2	Nonstructural protein 2
OAS	2′-5′-oligoadenylate synthetase
PKR	Protein kinase R
PML	Progressive multifocal leukoencephalopathy
PP1	Protein phosphatase 1
PRRs	Patter recognition receptors
RABV	Rabies virus
RIG-I	Retinoic acid-inducible gene I
RLRs	RIG-I-like receptors
RNase L	Ribonuclease L
SINV	Sindbis virus
siRNA	Small interfering RNA
STAT	Signal transducer and activator of transcription
STING	Stimulator of interferon genes
TBK1	TANK-binding kinase 1
TBEV	Tick-borne encephalitis virus
TAP	Transporter associated with antigen processing
TRIF	TIR-domain-containing adapter-inducing interferon-β
TRIM25	Tripartite motif-containing protein 25
TYK2	Tyrosine kinase 2
UL37	Unique long region protein 37
UL82	Unique long region protein 82
UL83	Unique long region protein 83
US2	Unique short protein 2
US3	Unique short protein 3
US6	Unique short protein 6
US11	Unique short protein 11
VEEV	Venezuelan equine encephalitis virus
VZV	Varicella-zoster virus
WNV	West Nile virus
WEEV	Western equine encephalitis virus
ZIKV	Zika virus
